# Supplementary material for: DMRforPairs: identifying Differentially Methylated Regions between unique samples using array based methylation profiles
Source: BMC Bioinformatics. 2014 May 15;15:141. doi: 10.1186/1471-2105-15-141 (PMC4046028; doi:10.1186/1471-2105-15-141)
Supplement: Additional file 2 — DMRforPairs output for the comparison of A431-MCF7 and NA17018-NA17105. Please start from the HTML files in each folder. Available via the BMC Bioinformatics website. [file 1471-2105-15-141-S2.zip › 1394847754114233_MOESM2_ESM/A431_MCF7/figures/33072.pdf]

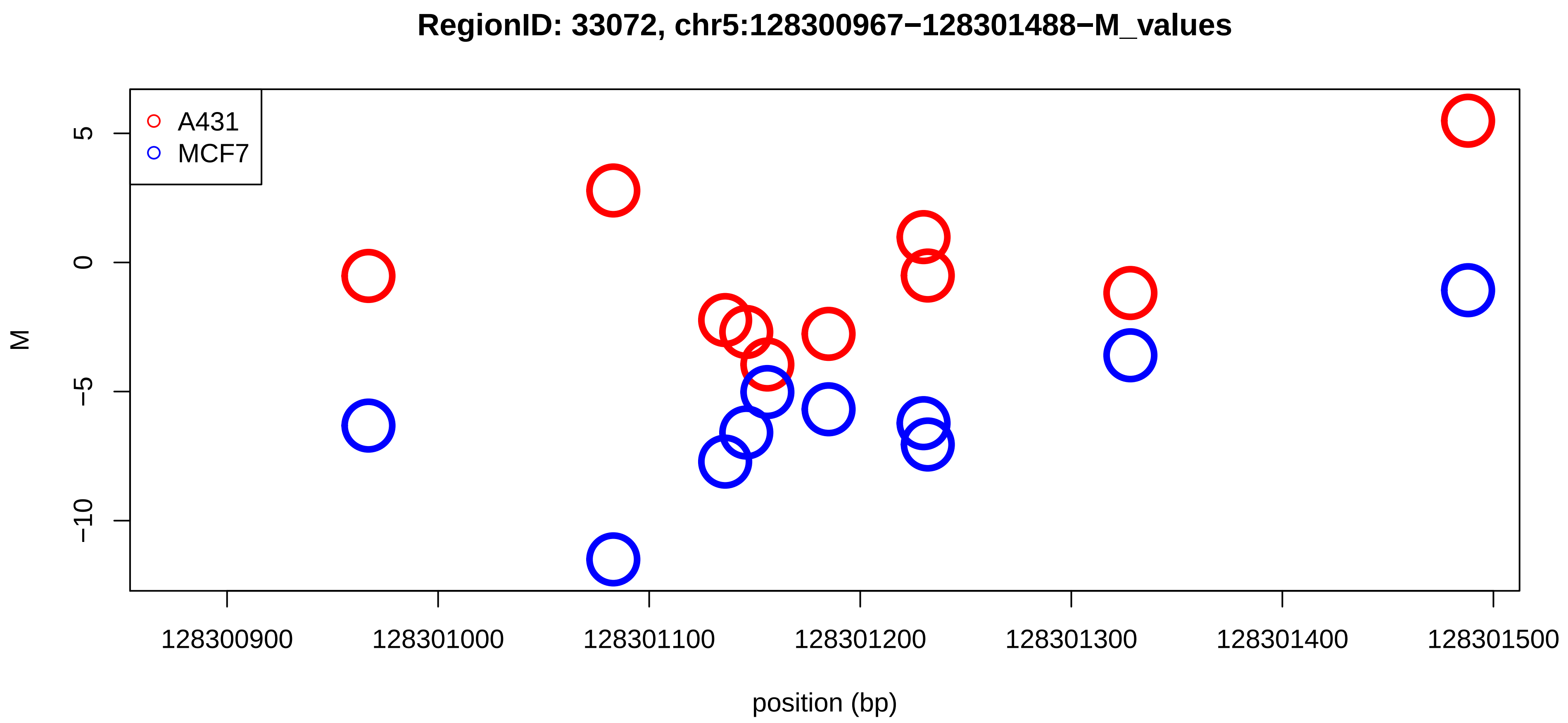

RegionID: 33072, chr5:128300967−128301488−M_values
A431
MCF7
M
5
0
−5
−10
128300900
128301000
128301100
128301200
128301300
128301400
128301500
position (bp)

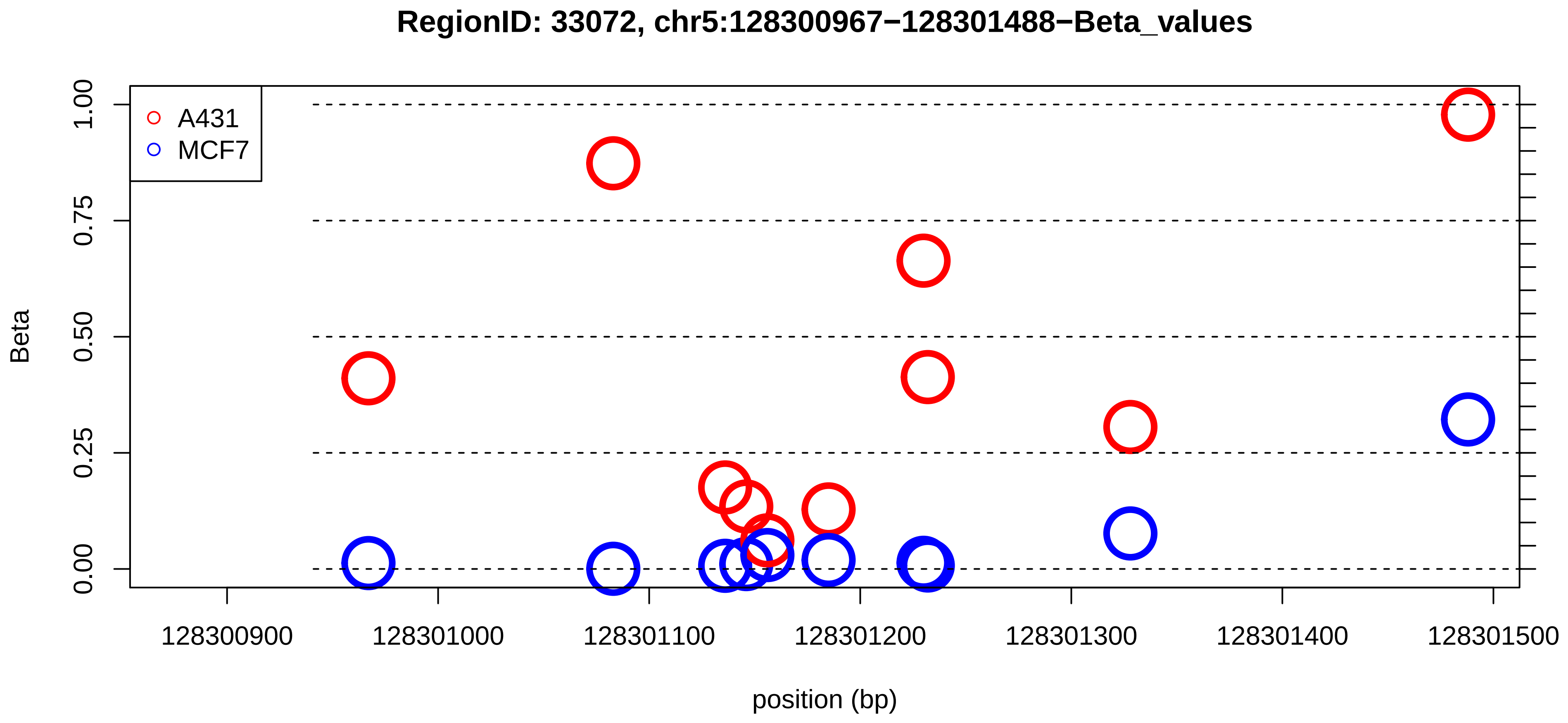

RegionID: 33072, chr5:128300967−128301488−Beta_values
A431
MCF7
Beta
1.00
0.75
0.50
0.25
0.00
128300900
128301000
128301100
128301200
128301300
128301400
128301500
position (bp)

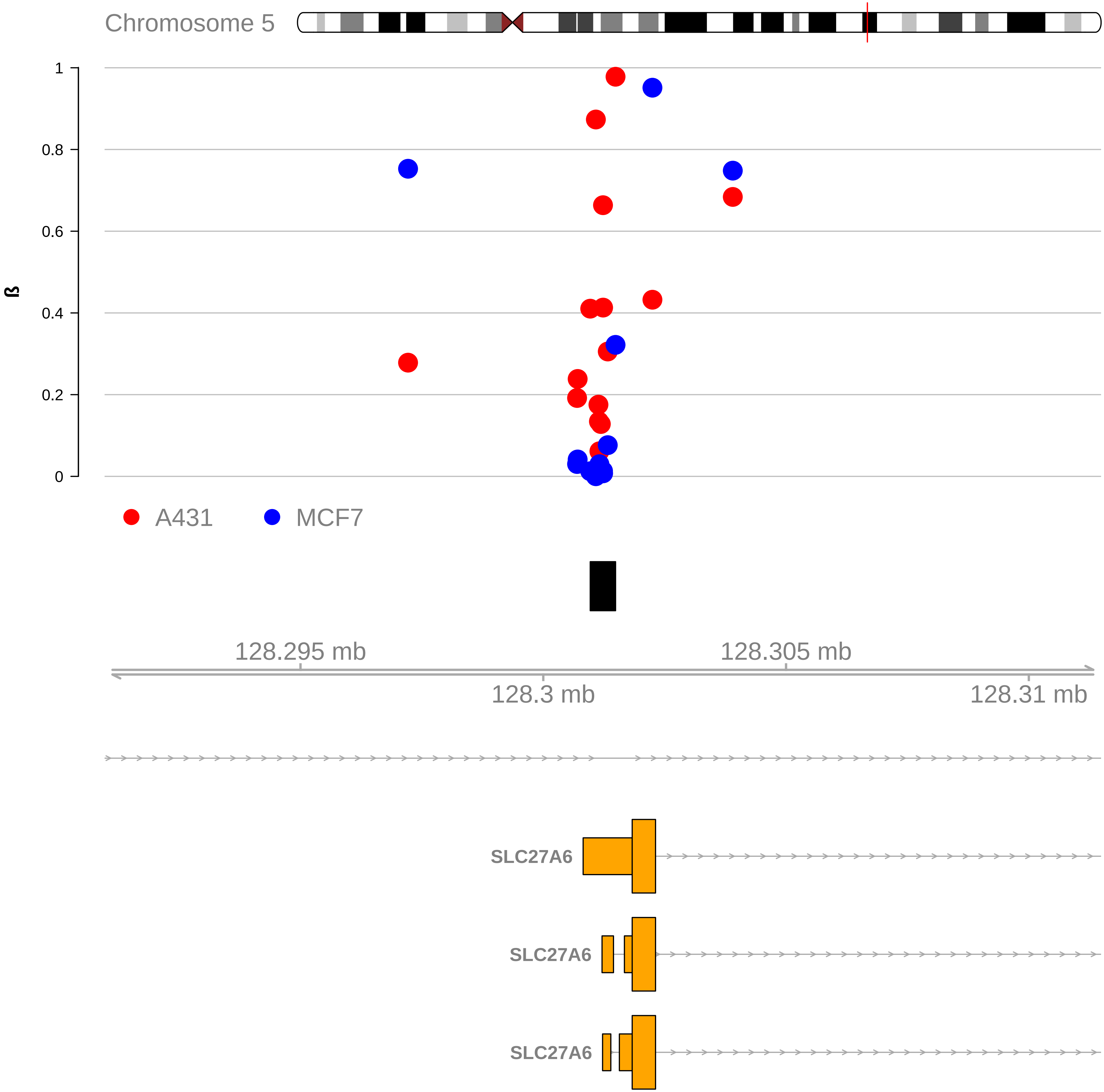

Chromosome 5
1
0.8
0.6
0.4
0.2
0
ß
A431
MCF7
128.295 mb
128.305 mb
128.3 mb
128.31 mb
SLC27A6
SLC27A6
SLC27A6
